# Effectiveness of Non-Pharmacological Interventions for Fatigue in Long COVID: A Systematic Review and Meta-Analysis of Randomized Controlled Trials

**DOI:** 10.3390/healthcare14203483

**Published:** 2026-10-16

**Authors:** Yanze Bi, Bo Yang, Siyu Chen, Qingrong Li, Rong Xu, Wenbo Zhang, Yunyi Yang, Wenshuang Zou, Shaodan Hu

**Affiliations:** 1 College of Integrated Chinese and Western Medicine, Changchun University of Traditional Chinese Medicine, Changchun 130117, China; 24211709050@ccucm.edu.cn (Y.B.); csy100865@163.com (S.C.); 15831075037@163.com (Q.L.); 19997066509@163.com (R.X.); 25211709064@ccucm.edu.cn (W.Z.); 25211709065@ccucm.edu.cn (Y.Y.); 2 Second Department of Pulmonary Diseases, Affiliated Hospital of Changchun University of Chinese Medicine, Changchun 130021, China; yangbofm@126.com; 3 Department of Gastroenterology, Affiliated Hospital to Changchun University of Traditional Chinese Medicine, Changchun 130021, China; zws1658@163.com; 4 Department of Pneumology, Affiliated Hospital of Changchun University of Traditional Chinese Medicine, Changchun 130021, China

**Keywords:** long COVID, non-pharmacological interventions, meta-analysis, randomized controlled trial

## Abstract

**Highlights:**

**What are the main findings?**

- Non-pharmacological interventions significantly reduced fatigue severity compared with control conditions [SMD = −0.43, 95% CI −0.68 to −0.18, *p* < 0.001]. Exercise training was associated with a statistically significant reduction in fatigue [SMD = −0.64, 95% CI −1.11 to −0.18, *p* = 0.006]. However, the difference in treatment effects between intervention modalities was not statistically significant (*p* for subgroup difference = 0.286). Intervention duration was identified as a statistically significant effect modifier: regimens lasting 8 weeks or longer produced greater improvements in fatigue relative to shorter intervention courses (between-subgroup difference *p* = 0.027).

**What are the implications of the main findings?**

- Non-pharmacological interventions may improve fatigue in adults with long COVID, but the magnitude of benefit varies across interventions and substantial heterogeneity remains. Exercise-based interventions showed a statistically significant pooled effect; however, there was no statistically significant evidence that exercise was superior to other intervention modalities. The findings regarding intervention duration should be considered exploratory because longer-duration interventions were disproportionately represented by exercise trials.
- Substantial between-study heterogeneity (*I*^2^ = 72.1%) and the paucity of randomised controlled trials evaluating non-exercise interventions underscore an urgent need for standardised intervention protocols and head-to-head comparative trials to establish optimal individualised rehabilitation regimens.

**Abstract:**

***Background***: Persistent fatigue is one of the most common and disabling symptoms of long COVID, yet no pharmacological treatments are currently established. Non-drug strategies are increasingly used, but evidence directly comparing these approaches remains limited. This systematic review and meta-analysis evaluated the effects of non-pharmacological interventions on fatigue in adults with long COVID and explored potential sources of heterogeneity. ***Materials and Methods***: Following PRISMA 2020 and the Cochrane Handbook, we searched four databases from inception to December 2025 for RCTs comparing non-pharmacological interventions with control conditions in adults with WHO-defined long COVID. The primary outcome was pre-specified as fatigue severity; no secondary outcomes were pre-specified. Methodological quality was assessed using the RoB 2 tool. Random-effects models were used to pool SMDs, with exploratory subgroup, sensitivity, and publication bias analyses. ***Results***: Thirteen RCTs (1276 participants) were included. Non-pharmacological interventions significantly reduced fatigue [SMD = −0.43, 95% CI: −0.68 to −0.18, *p* < 0.001], with substantial heterogeneity (*I*^2^ = 72.1%). Subgroup analyses showed that exercise training yielded significant benefit [SMD = −0.64, 95% CI: −1.11 to −0.18, *p* = 0.006], whereas cognitive behavioural therapy, non-invasive neuromodulation, and nutritional supplementation did not reach statistical significance. Interventions lasting ≥8 weeks were associated with greater improvement (between-subgroup *p* = 0.027). Sensitivity analyses confirmed robustness, and no publication bias was detected. ***Conclusions***: Non-pharmacological interventions, particularly exercise-based programmes, are associated with reduced fatigue in people with long COVID, with interventions lasting ≥8 weeks showing greater average improvements. Exercise-based rehabilitation currently has the most consistent evidence base among the evaluated modalities, although the difference in effects between intervention modalities was not statistically significant (*p* = 0.286). Further high-quality trials are required to determine the comparative effectiveness of emerging approaches and to identify optimal treatment strategies.

## 1. Introduction

The World Health Organization (WHO) defines long COVID as symptoms that develop 3 months or more after the onset of confirmed or suspected SARS-CoV-2 infection, persist for at least 2 months, and cannot be explained by an alternative diagnosis [1]. Epidemiological data indicate that approximately 43% of individuals infected with SARS-CoV-2 develop long COVID, with prevalence rates of 54% among patients hospitalised during acute infection and 34% among non-hospitalised patients [2]. However, these estimates vary across studies because of differences in case definitions, follow-up periods, and population characteristics, highlighting the substantial heterogeneity in the reported burden of long COVID. The condition impairs both physical and mental health and places a considerable burden on healthcare systems worldwide [3].

Long COVID presents with a wide spectrum of persistent and fluctuating symptoms; more than 200 distinct manifestations have been reported to date, involving multiple organ systems including the respiratory, circulatory, neurological, immune and endocrine systems [4]. Fatigue is one of the most common and long-lasting symptoms, and substantially impairs patients’ daily function and quality of life [5]. The incidence of fatigue among patients with long COVID ranges from 26.9% to 30.7%, although estimates may vary according to follow-up duration and population characteristics. In some cases, severe fatigue can persist for 12 months or longer after acute infection [6,7]. Older age, female sex, and hospitalisation during acute illness are established risk factors for persistent fatigue in this population [8,9].

Although fatigue is often considered a predominantly physical symptom, increasing evidence suggests that long COVID-related fatigue is a complex and multidimensional condition involving both physical and cognitive dysfunction. Patients frequently experience accompanying cognitive impairment, attentional deficits, executive dysfunction, and reduced dual-task performance, which may further impair functional recovery and daily activities [10]. Therefore, rehabilitation approaches targeting multiple domains rather than fatigue alone have attracted increasing clinical interest. Recent pilot studies have explored innovative rehabilitation strategies, including augmented reality-based dual-task rehabilitation and targeted physiological rehabilitation, suggesting potential benefits for patients with long COVID experiencing fatigue and cognitive impairment [11,12]. However, these approaches remain preliminary and require further validation through well-designed randomized controlled trials.

Long COVID-related fatigue may also coexist with exercise intolerance and post-exertional symptom exacerbation (PESE), in which physical or cognitive exertion can lead to the worsening of fatigue and other symptoms [13,14]. These features further illustrate the clinical heterogeneity of long-COVID fatigue and highlight the importance of individualized, symptom-guided rehabilitation rather than uniform exercise prescriptions [15].

Currently, no specific pharmacotherapy is approved for long-COVID fatigue, and conventional treatments provide limited clinical benefit [16]. Non-pharmacological interventions, given their favourable safety profile and broad accessibility, are widely used as alternative and adjunctive strategies for managing this symptom [17]. Previous systematic reviews have evaluated several non-pharmacological approaches for long COVID-related fatigue. Exercise-based rehabilitation and pulmonary rehabilitation have shown potential benefits for fatigue and functional outcomes, while broader reviews have also considered cognitive behavioural and other rehabilitation strategies [16,18,19,20,21]. Among these approaches, exercise-based rehabilitation currently has the most consistent evidence supporting improvements in fatigue. However, the current evidence base still has several gaps. First, most studies focus on a single intervention modality, and no systematic review to date has comprehensively compared the efficacy of multiple non-pharmacological approaches [22]. Second, intervention protocols vary substantially across studies, and the sources of this heterogeneity have not been systematically explored, limiting the generalisability of existing findings. Finally, a growing number of high-quality randomized controlled trials (RCTs) have been published in recent years, yet these have not been incorporated into existing evidence syntheses, leaving prior conclusions potentially outdated.

Against this background, we classified the eligible non-pharmacological interventions into four categories according to their primary therapeutic approach: exercise training, cognitive behavioural therapy, non-invasive neuromodulation, and nutritional supplementation, with detailed definitions provided in the Supplementary Materials (File S2). Using a PICO framework, this review evaluated adults with long COVID (Population) receiving these non-pharmacological interventions (Intervention), compared with usual care, waitlist, sham/placebo, or no intervention (Comparison), with fatigue severity assessed using validated instruments as the primary outcome (Outcome). Exploratory subgroup analyses were conducted according to intervention type, intervention duration, and fatigue assessment instrument to investigate potential sources of heterogeneity. Our aim was to provide updated and more comprehensive evidence to inform the clinical management of long-COVID fatigue.

## 2. Materials and Methods

We conducted this systematic review and meta-analysis in accordance with the PRISMA 2020 checklist [23] (File S1), The review protocol was prospectively registered in PROSPERO (CRD420251268975) on 18 *December* 2025, with the review start date recorded as 18 *December* 2025. Subgroup analyses were not pre-specified in the registered protocol and were therefore considered exploratory.

### 2.1. Search Strategy

Two researchers (Yanze Bi and Siyu Chen) comprehensively searched PubMed, Embase, the Cochrane Library, and Web of Science from database inception to 18 *December* 2025. The search was conducted between 1 *December* and 18 *December* 2025. The search strategy incorporated both MeSH headings and text words, such as “Post-Acute COVID-19 Syndrome”, “Fatigue”, along with free terms including “Long COVID”, “Long Haul COVID-19”, “Lassitude”, and “randomized controlled trial”. The full search strategies for all databases are available in File S4.

### 2.2. Study Selection

Studies were eligible for inclusion if they met the following criteria: (1) Population: age ≥ 18 years, with a diagnosis of long COVID consistent with the WHO definition; for studies conducted before the WHO definition was established, eligibility was assessed according to the study authors’ definition of persistent post-COVID symptoms, provided that the reported population was clinically consistent with the long COVID population targeted by this review. Studies with mixed populations were eligible only when data for participants meeting the predefined long COVID criteria could be separately extracted; (2) Intervention: the experimental group received non-pharmacological interventions, including exercise training, cognitive behavioural therapy, nutritional supplements, or non-invasive neuromodulation, with no restrictions on the form, frequency, or duration of the intervention. For interventions containing multiple therapeutic components, the intervention was classified according to its predominant therapeutic component; interventions combining components from different predefined categories were considered multimodal and were handled according to the prespecified intervention classification criteria described in File S2; (3) Comparison: the control group received an eligible comparator, including placebo, usual care, waitlist, or no intervention; (4) Outcome: the pre-specified primary outcome was fatigue severity, assessed using any validated fatigue assessment scale, with extractable continuous outcome data (mean and standard deviation or equivalent statistics) reported. No additional outcomes were pre-specified; (5) Study design: randomized controlled trials (RCTs). Detailed definitions of each intervention modality are presented in File S2.

Studies were excluded if they: (1) did not include adults with a clinically relevant long COVID population; (2) did not evaluate an eligible non-pharmacological intervention; (3) lacked an eligible comparator; (4) did not assess fatigue using a validated instrument; (5) did not provide sufficient data for quantitative synthesis; or (6) were not eligible study designs, including observational studies, case reports, reviews, protocols, conference abstracts without sufficient data, and other non-primary research reports. When multiple publications originated from the same underlying trial, the trial was treated as a single study to avoid double-counting participants.

**Figure 1:**
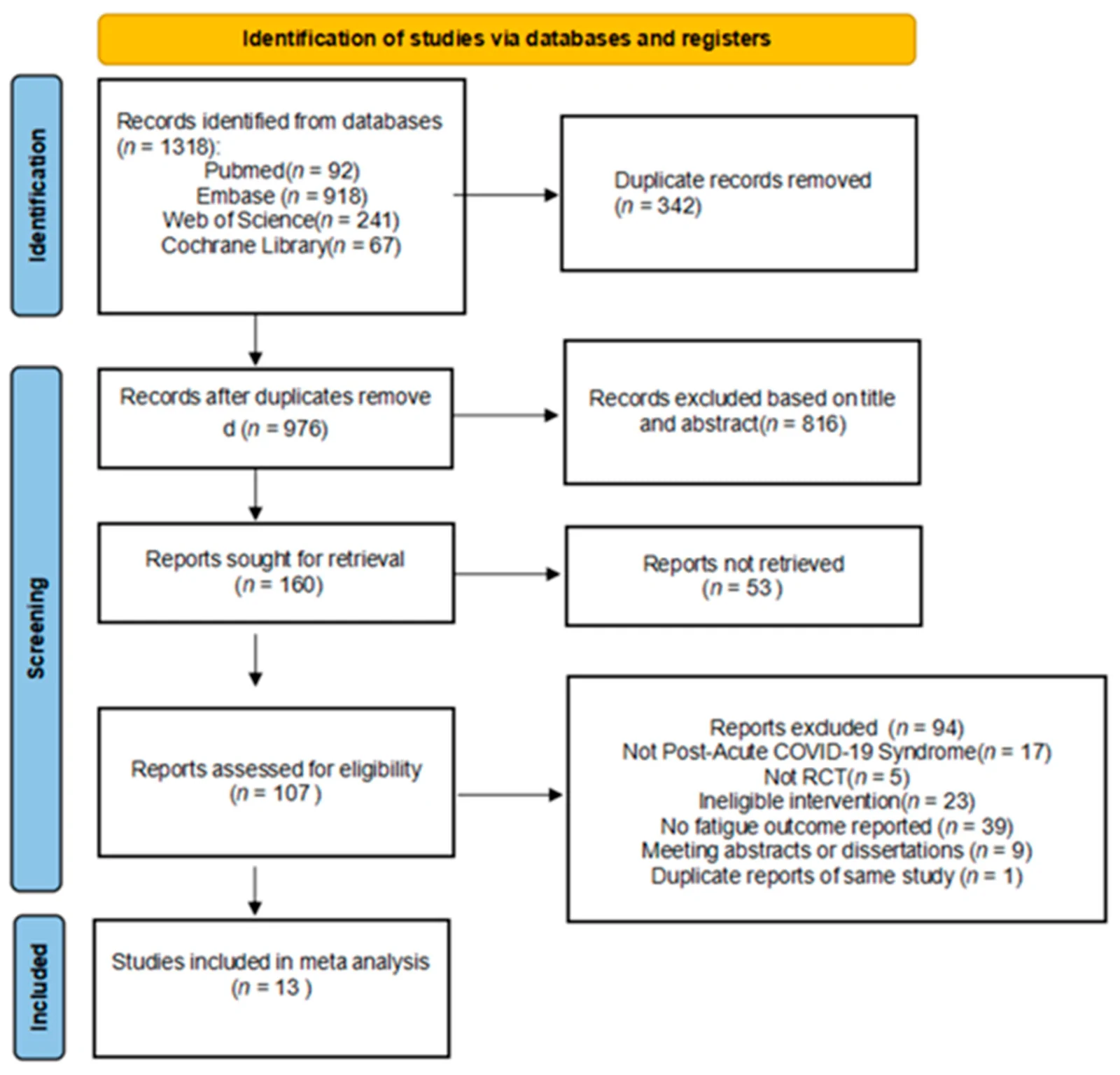
PRISMA flowchart of the study selection process.

All retrieved citations were managed in EndNote X9 (Clarivate, Philadelphia, PA, USA), and duplicates were excluded. Two independent reviewers (Yanze Bi and Siyu Chen) screened the records following the PRISMA workflow. Initial screening of titles and abstracts served to exclude clearly ineligible studies, after which the full texts of candidate studies were retrieved and assessed against the eligibility criteria. No automated or machine-learning screening tool was used; all title/abstract and full-text screening was performed manually by the two independent reviewers. Disagreements between reviewers were resolved by discussion or through consultation with a third reviewer (Shaodan Hu). The screening procedure is outlined in the PRISMA diagram (Figure 1).

### 2.3. Data Extraction

Data extraction was performed independently by two reviewers following a pre-specified coding template. The data extraction form was piloted before formal data extraction and refined as necessary. Data extraction was then performed independently by two reviewers using the pre-specified coding template. The extracted information encompassed: (1) study provenance (first author, year, and setting); (2) participant demographics (sample size, sex ratio, mean age, time since infection, baseline fatigue, adherence, adverse events, comorbidities, hospitalization status, and baseline fatigue severity measured with validated instruments); (3) intervention parameters (modality, duration, and programme content); (4) control conditions; and (5) fatigue outcomes (post-intervention fatigue scores were used as the primary effect estimates). Studies that did not provide sufficient extractable data were excluded to maintain the integrity of the meta-analysis. Inter-reviewer disagreements were resolved by consensus.

The extracted information on interventions and main outcomes is summarised in Table 1.

### 2.4. Risk-of-Bias Assessment

Risk of bias in each included RCT was independently assessed by two reviewers using the Cochrane Risk of Bias 2 (RoB 2) tool [24]. The assessment covered five domains: randomisation process, deviations from intended interventions, missing outcome data, measurement of the outcome, and selection of the reported result. Risk of bias judgments were made at the outcome level, with domain-level assessments informing the overall risk-of-bias judgment for each outcome. Studies were subsequently assigned an overall risk-of-bias rating of low, some concerns, or high. Inter-rater agreement between the two reviewers was assessed using Cohen’s kappa coefficient. Any disagreements between the two reviewers were resolved through discussion and consensus, with consultation of a third reviewer when necessary.

For the measurement-of-the-outcome domain, particular consideration was given to the subjective nature of fatigue assessment and the potential influence of knowledge of intervention assignment. The use of a validated fatigue instrument was not considered sufficient by itself to indicate low risk of bias when concerns regarding outcome measurement or lack of blinding were present.

### 2.5. Statistical Analysis

It should be acknowledged that the included non-pharmacological interventions, including exercise training, cognitive behavioural therapy, non-invasive neuromodulation, and nutritional supplementation, are heterogeneous in their therapeutic mechanisms. However, these interventions share a common clinical objective of improving fatigue-related outcomes in individuals with long COVID. Therefore, the primary meta-analysis was conducted to provide an overall estimate of the effects of non-pharmacological interventions on fatigue.

All pooled analyses were conducted in Stata 17.0 (StataCorp LLC, College Station, TX, USA). Given that all outcomes were continuous, the effect size was expressed as *Hedges’ g* (a bias-corrected SMD) for studies using different fatigue assessment tools, and as the mean difference (MD) for studies using identical instruments. Effect estimates are reported alongside their 95% CIs. For studies reporting more than one fatigue scale measuring the same underlying construct, the outcome measures were combined to generate a single study-level effect estimate, so that each study contributed only one effect size to the primary meta-analysis. Means were combined by averaging the scale means. Standard deviations were combined using the Cochrane Handbook formula for combining outcomes from the same study, which requires an assumed correlation between the measures. A correlation of r = 0.7 was assumed for this purpose. This assumption was used only for variance calculation and did not affect the point estimate of the combined mean. When multiple post-intervention assessment time points were available, only the assessment obtained immediately after completion of the full planned intervention period was included in the primary meta-analysis. Intermediate assessments and later follow-up assessments were excluded from the primary analysis to maintain a consistent definition of the post-intervention time point across studies.

The dataset included one three-arm RCT among the 13 eligible studies. To circumvent unit-of-analysis error resulting from repeated use of the same control group, we divided the control arm’s sample size equally between the two active comparisons—as recommended by the Cochrane Handbook—leaving the mean and SD of the control group intact [25]. This yielded 14 distinct intervention–control pairs, which explains why subgroup analyses may report more comparisons than the total number of included trials.

We assessed statistical heterogeneity by means of Cochran’s Q and the *I*^2^ index, with the significance threshold set at α = 0.05. Values of *I*^2^ below 50% suggested low-to-moderate heterogeneity and led to the use of a fixed-effect inverse-variance model; values of 50% or above were considered indicative of substantial heterogeneity, for which a random-effects model with the DerSimonian–Laird approach was applied [26]. A two-sided *p* < 0.05 was accepted as statistically significant, and the results are visualised with forest plots.

### 2.6. Subgroup Analysis and Sensitivity Analysis

Stratification by intervention type, duration, and fatigue rating scale was applied in exploratory subgroup analyses to investigate potential sources of heterogeneity. Differences in pooled effect sizes between subgroups were tested using the between-group Q statistic within a mixed-effects model framework [27,28], with *p* < 0.05 considered statistically significant.

Sensitivity analysis was performed using the leave-one-out method, in which the pooled effect size was recalculated iteratively by omitting one study at a time to assess the influence of each individual study on the overall estimate and verify the robustness of the results.

Funnel plot asymmetry was evaluated visually, and Egger’s regression test was used to statistically detect publication bias. To further adjust the summary effect estimate for potential bias, we employed the trim-and-fill technique. These publication bias assessments were performed only for outcomes with at least 10 included studies, as statistical power for detecting funnel plot asymmetry is insufficient with smaller study sets [29,30,31].

**Table 1:** Characteristics of the studies included in the systematic review and meta-analysis.

Authors (Country)	Group	Mean Age (Years)	M/F	Duration	Intervention		Comparison	Outcome Measure
Anne-Lotte Redel et al., 2024 [32] (The Netherlands)	**IG:36** **CG:36**	**NA**	**27/45**	**6 weeks**	**Nutritional intervention** **:** **600 mg** **lactoferrin capsules twice a day**		**Placebo**	**FAS**
Monica Busse et al., 2025 [33] (UK)	**IG:270** **CG:274**	**50.0**	**143/394**	**12 weeks**	**CBT: The Listen intervention offered up to six one-to-one personalised self-management sessions (minimum three), delivered remotely via video or telephone (≤1 h each) by trained healthcare practitioners, with a supporting handbook.**		**Usual care**	**FIS**
Arno Kerling et al., 2024 [34] (Germany)	**IG:30** **CG:32**	**46.4**	**20/42**	**12 weeks**	**Exercise: Weekly physical activity consisted of 150 min of moderate-intensity exercise (at 60–75% of the maximum heart rate measured during the incremental exercise test). In addition, once a week, a higher-intensity exercise session was scheduled, such as 3–10 min of stair climbing or 1–3 min of sit-to-stand exercises, to allow patients to exceed their exercise heart rate limit if tolerated. Patients performed the training independently at home.**		**No intervention**	**FAS**
Andreas Barz et al., 2024 [35] (Germany)	**IG:58** **CG:60**	**53.5**	**37/81**	**10 weeks**	**Exercise: 1–3 times per week, consisting of both resistance training and aerobic training. Resistance training focuses on whole-body exercises, while aerobic training can be performed in various forms (e.g., cycle ergometer, treadmill, etc.). The exercise mode, intensity, and volume are all tailored to the individual’s circumstances.**		**Waitlist**	**FSS**
Alper Percin et al., 2025 [36] (Türkiye)	**IG:21** **CG:21**	**NA**	**20/22**	**20 days**	**N** **on-** **invasive neuromodulation: Once daily, 30 min per session. The** **Vagustim device was used for electrical stimulation. The electrical stimulation with a frequency of biphasic continuous 10 hertz (Hz) and a pulse width of 300 μs was applied to the participants. Suprathreshold intensities were adjusted between 0.5 and 12 mA for each patient. The electrodes were placed in the tragus and concha regions after electrode gel had been applied to them.**		**Placebo**	**FSS**
Tanja A. Kuut et al., 2023 [37] (The Netherlands)	**IG:57** **CG:57**	**NA**	**31/83**	**19 weeks**	**CBT: Patients received an average of 11.8 interactions through CBT developed specifically for post-COVID-19 fatigue. They could access treatment modules online or opt for face-to-face sessions.**		**Usual care**	**CIS-fatigue**
Johanna Sick et al., 2024 [38] (Austria)	**IG1:14** **IG2:14** **CG:14**	**41.2**	**9/33**	**12 weeks**	**Exercise: 3 times per week. The endurance training group performed pure aerobic training (cycle ergometer, elliptical, or treadmill). In weeks 1–2, each session lasted 30 min; from weeks 3–12, the program consisted of two sessions at VT1 intensity and one interval session per week, with total duration gradually increasing up to a maximum of 60 min per session. The concurrent training group combined resistance training and aerobic training; resistance intensity was prescribed using RPE (6–20 Borg scale), while the aerobic component was identical to that of the endurance group.**		**No intervention**	**FSS**
Mohammad Keilani et al., 2025 [39] (Austria)	**IG:10** **CG:10**	**NA**	**9/11**	**5 weeks**	**Non-invasive neuromodulation: The patients received 10 sessions of PEMF twice weekly for 5 weeks. Each session lasted 30 min. the Papimi™ Delta professional electromagnetic field therapy device was used for treatment**. **The pulse rate was 2.5 Hz in the dorsal area and 1 Hz for all other locations.**		**Placebo**	**BFI** **,** **MFI**
Tess Volckaerts et al., 2025 [40] (Belgium)	**IG:39** **CG:37**	**NA**	**27/49**	**12 weeks**	**Exercise: Three times per week, 30 min per session. The programme included the following components: information about long COVID, a healthy lifestyle and behaviour change towards a more active lifestyle, endurance training, strength training, breathing exercises and inspiratory muscle training**		**Usual care**	**CIS-fatigue**
Yuanyuan Chen et al., 2025 [41] (China)	**IG:55** **CG:55**	**37.0**	**41/69**	**12 weeks**	**Nutritional intervention** **: 1.6 g daily Cordyceps sinensis mycelium culture extract (Cs4)**		**Waitlist**	**BFI**
Alberto Melián-Ortíz et al., 2025 [42] (Spain)	**IG:9** **CG:7**	**NA**	**0/16**	**8 weeks**	**N** **on-invasive neuromodulation** **: Three sessions per week, 60 min per session. The treatment delivered targeted central autonomic nervous system modulation using directional electrodes positioned over the C7 cervical vertebra, with stimulation intensity set at 3 V (low level).**		**Placebo**	**MFIS**
Zeliha Çelik et al., 2024 [43] (Türkiye)	**IG:14** **CG:13**	**NA**	**5/22**	**6–7 weeks**	**Exercise: A total of 20 individualized counseling sessions were delivered, at least 3 days per week, each session lasting 15–20 min. Through online counseling (by telephone or computer video call), participants received information about COVID-19, physical activity, and the basics of exercise. An individualized exercise program was developed, which included aerobic exercise (walking/cycling, at 60–80% of maximum heart rate, 30 min per session, at least 3 days/week), calisthenic exercises, and upper and lower limb resistance training (using elastic bands/sandbags, 3 sets of 10 repetitions, at RPE 7, at least 3 days/week).**		**No intervention**	**FSS**
Monika Klírová et al., 2024 [44] (Czech Republic)	**IG:16** **CG:17**	**NA**	**10/23**	**4 weeks**	**N** **on-invasive neuromodulation** **: A total of 20 sessions, 30 min per session. Transcranial direct current stimulation (tDCS) was delivered using a HDCStim programmable stimulator (Newronica, Cologno Monzese, Italy) in a double-blind design. The stimulating electrodes were positioned with the anode over the F3 area (according to the international 10–20 EEG system) and the cathode over the contralateral F4 area. Saline-soaked sponge electrodes were secured to the scalp using a Mind-cap, with placement corresponding to the left and right dorsolateral prefrontal cortex (DLPFC), respectively.**		**Placebo**	**FIS**
**Authors** **(Country)**	**B** **aseline fatigue**		**A** **dherence**	**Adverse events**	**T** **ime since infection**	**Comorbidities**	**H** **ospitalization**	
Anne-Lotte Redel et al., 2024 [32] (The Netherlands)	**IG: 33.5** **CG: 33.5**		**NA**	**IG: 8/36, 22.2%** **CG: 9/36, 25%**	**IG: 204.5 (121.3–320.8)** **CG: 183.7 (134.3–337.0)**		**Hospital admission during acute infection: Lactoferrin 5 (13.9%); Placebo 2 (2.8%).**	
Monica Busse et al., 2025 [33] (UK)	**IG: 92.7 ± 35.3;** **CG: 89.3 ± 35.1.**		**At least 3 sessions: 212/270 (78.5%)**	**None of the reported serious adverse events was considered related to the intervention.**	**NA**	**NA**	**NA**	
Arno Kerling et al., 2024 [34] (Germany)	**IG: 35.1 ± 7.4;** **CG: 35.6 ± 7.4**		**NA**	**NA**	**NA**	**NA**	**NA**	
Andreas Barz et al., 2024 [35] (Germany)	**IG: 5.67 ± 0.92;** **CG:5.49 ± 0.87**		**NA**	**Two participants discontinued the exercise intervention due to transient worsening of fatigue**	**≥12 weeks**	**NA**	**Exclusion criteria were… hospitalization due to COVID-19.**	
Alper Percin et al., 2025 [36] (Türkiye)	**IG: 5.57 ± 1.20** **CG: 5.16 ± 1.71**		**NA**	**NA**	**at least 12 weeks**	**NA**	**NA**	
Tanja A. Kuut et al., 2023 [37] (The Netherlands)	**IG:47.8** **CG:47.0**		**NA**	**IG: 7/57, 12%** **CG: 14/57, 25%**	**IG:** **191.2 (75.8)** **CG:** **183.7 (75.5)**	**IG:** **None:32 (56%)** **1: 18 (32%)** **2 or more:** **7 (12%))** **CG:** **None: 31 (54%)** **1:** **17 (30%)** **2 or more:** **9 (16%)**	**Did not require hospitalization during their initial COVID-19 (101/114 [89%]**	
Johanna Sick et al., 2024 [38] (Austria)	**IG1: 4.7 ± 1.2;** **IG2: 4.5 ± 0.8;** **CG: 4.8 ± 1.2.**		**Amongst the intervention groups, adherence to the training sessions was at 92.6% on average**	**2 participants of the training groups stopped due to a worsening of their symptoms in response to the intervention.**	**Days post-infection: Total 265 (180)**	**NA**	**Exclusion criteria were hospitalization during acute COVID-19**	
Mohammad Keilani et al., 2025 [39] (Austria)	**BFI:** **IG: 5.16 ± 1.39;** **CG: 6.12 ± 1.55** **MFI:** **IG: 72.9 ± 13.7; CG: 74.8 ± 9.22.**		**IG: 95%** **CG: 70%**	**In the intervention group** **…** **three patients were tired, two patients reported that they had headache, and one patient had muscle soreness. In the control group, two patients reported tiredness, one patient had vertigo** **…**	**IG:** **278 (205.75)** **CG:** **277.5 (183)**	**Comorbidities: Musculoskeletal disease, Hypertension, Cardiovascular diseases, Gastrointestinal diseases, Thyroid gland diseases, Pulmonary diseases, Adiposity, Others**	**NA**	
Tess Volckaerts et al., 2025 [40] (Belgium)	**IG: 45.1 ± 7.7; CG: 48.2 ± 5.6.**		**Overall adherence** **:** **81%**	**There were eight AEs (four PEM, two musculoskeletal issues, one nausea and one atypical chest pain) and one unrelated SAE (Staphylococcus epidermidis sepsis)**	**IG: 366** **(189–633)** **CG:** **2** **82** **(196–477)**	**Charlson Comorbidity Index (total score): Intervention 1 (0–2); Control 1 (0–2)**	**Hospitalisation during acute COVID-19 infection, *n* (%): Intervention 3 (7.7%); Control 3 (8.1%).**	
Yuanyuan Chen et al., 2025 [41] (China)	**IG:46.6 ± 15.1;** **CG: 46.4 ± 16.6.**		**Study compliance, *n* (%): Taking 100% of study products: Cs4 46 (0.84); Control 40 (0.73).**	**Adverse events** **…** **AST: Cs4 4 (7.3%), Control 1 (1.8%); ALT: Cs4 3 (5.4%), Control 0 (0%)** **…** **None of the adverse events were likely to be associated with the study products. Adverse events** **…** **AST: Cs4 4 (7.3%), Control 1 (1.8%); ALT: Cs4 3 (5.4%), Control 0 (0%)** **…** **None of the adverse events were likely to be associated with the study products.**	**median: 5.0 [2.0, 9.0]**	**NA**	**PCFS score, *n* (%): 2: 109 (99.1%).**	
Alberto Melián-Ortíz et al., 2025 [42] (Spain)	**IG: 60.67 ± 18.36** **CG: 62.71 ± 9.41**		**NA**	**NA**	**NA**	**NA**	**NA**	
Zeliha Çelik et al., 2024 [43] (Türkiye)	**IG: 3.89** **CG: 3.78**		**NA**	**NA**	**Individuals in the persistent period (>12 weeks)**	**NA**	**NA**	
Monika Klírová et al., 2024 [44] (Czech Republic)	**IG: 77.2 ± 28.5; CG: 101.6 ± 25.2.**		**NA**	**During the initial 2 weeks** **…** **eight of the sixteen patients (50%) receiving active tDCS and nine of the seventeen (53%) patients assigned to the sham tDCS group experienced mild to moderate side effects such as burning or tingling.**	**Months since COVID-19: Active 12.2 (8.3); Sham 14.4 (7.0)**	**Hypertension, Diabetes mellitus type 2, Dyslipidemia, Hypothyroidism, History of psychiatric illness.**	**NA**	

**Abbreviations**: IG: Intervention group; CG: Control group; M: Male; F: Female; NA: Not reported; CBT: Cognitive behavioural therapy; FAS: Fatigue Assessment Scale; FIS: Fatigue Impact Scale; FSS: Fatigue Severity Scale; CIS-fatigue: Checklist Individual Strength-fatigue subscale; BFI: Brief Fatigue Inventory; MFI: Multidimensional Fatigue Inventory; MFIS: Modified Fatigue Impact Scale.

## 3. Results

### 3.1. Search Results

We identified 1318 records through database searching and imported them into EndNote X9 for management. After removing 342 duplicates, 976 records were screened based on titles and abstracts, of which 816 were excluded. Of the remaining 160 reports sought for retrieval, 53 could not be obtained. The remaining 107 reports were assessed for full-text eligibility, of which 94 were excluded for the following reasons: not post-acute COVID-19 syndrome (*n* = 17), not RCT (*n* = 5), ineligible intervention (*n* = 23), no fatigue outcome reported (*n* = 39), meeting abstracts or dissertations (*n* = 9), duplicate reports of the same study (*n* = 1), and reviews, leaving 13 studies eligible for qualitative synthesis and meta-analysis. The PRISMA flow diagram detailing the selection process is presented in Figure 1.

### 3.2. Study Characteristics

Table 1 summarizes the baseline characteristics of the 13 included randomized controlled trials (RCTs). A total of 1276 patients were enrolled, with 643 in the intervention groups and 633 in the control groups. Geographically, the studies were conducted across 9 countries: two were conducted in each of The Netherlands, Austria, Türkiye, and Germany, and one each in the UK, Belgium, China, Czech Republic and Spain. All participants were adults (aged ≥18 years). Five studies reported the mean age of participants, ranging from 37.0 to 53.5 years, while the remaining 8 studies did not report detailed age statistics. All studies documented gender distribution: 379 males, 890 females, and 7 participants identified as other genders. Twelve studies recruited mixed-gender samples, with the proportion of females ranging from 52.4% to 81.5%, and one study exclusively enrolled female participants. The duration of long COVID at the baseline varied across studies, with median or mean times since infection ranging from 5 months to approximately 12 months. Hospitalisation rates during acute COVID-19 were generally low, as most trials recruited non-hospitalised participants; among studies reporting this information, the proportion hospitalised ranged from 0% to 13.9%. Baseline fatigue severity, adherence rates, adverse events, and comorbidities were extracted when reported and are summarised in Table 1. However, reporting of these variables was inconsistent across studies, and they were not incorporated into quantitative analyses due to insufficient data.

In terms of study design, 12 trials adopted a two-arm design and 1 trial used a three-arm design. The intervention groups received a diverse range of non-pharmacological interventions. To facilitate subgroup analyses, these interventions were categorized into four types based on their therapeutic mechanisms: (1) exercise training; (2) cognitive behavioural therapy; (3) non-invasive neuromodulation; (4) nutritional intervention. The intervention frequency ranged from once weekly to once daily, and the intervention duration spanned from 2.9 to 19 weeks. Control groups received usual care, waitlist, placebo, sham control, or no intervention. Intervention implementation varied across studies in terms of delivery format, treatment frequency, duration, intensity, and degree of supervision. Exercise interventions included supervised, remotely guided, and home-based programmes, while other modalities used structured behavioural sessions, standardized neuromodulation protocols, or fixed nutritional supplementation regimens, as detailed in Table 1. Adherence, provider qualifications, monitoring procedures, and adverse events were not consistently reported across the included studies and therefore could not be systematically extracted or summarized.

All included studies used validated fatigue assessment tools, including the Fatigue Assessment Scale (FAS), the Fatigue Impact Scale (FIS), the Fatigue Severity Scale (FSS), the fatigue subscale of the Checklist Individual Strength (CIS-fatigue), the Brief Fatigue Inventory (BFI), the Multidimensional Fatigue Inventory (MFI), and the Modified Fatigue Impact Scale (MFIS). Given the inconsistent scoring scales across instruments, mean difference (MD) or standardized mean difference (SMD) was applied for pooling effect sizes depending on whether the same assessment tool was used across studies, as detailed in the Section 2.5.

### 3.3. Risk of Bias Assessment Results

**Figure 2:**
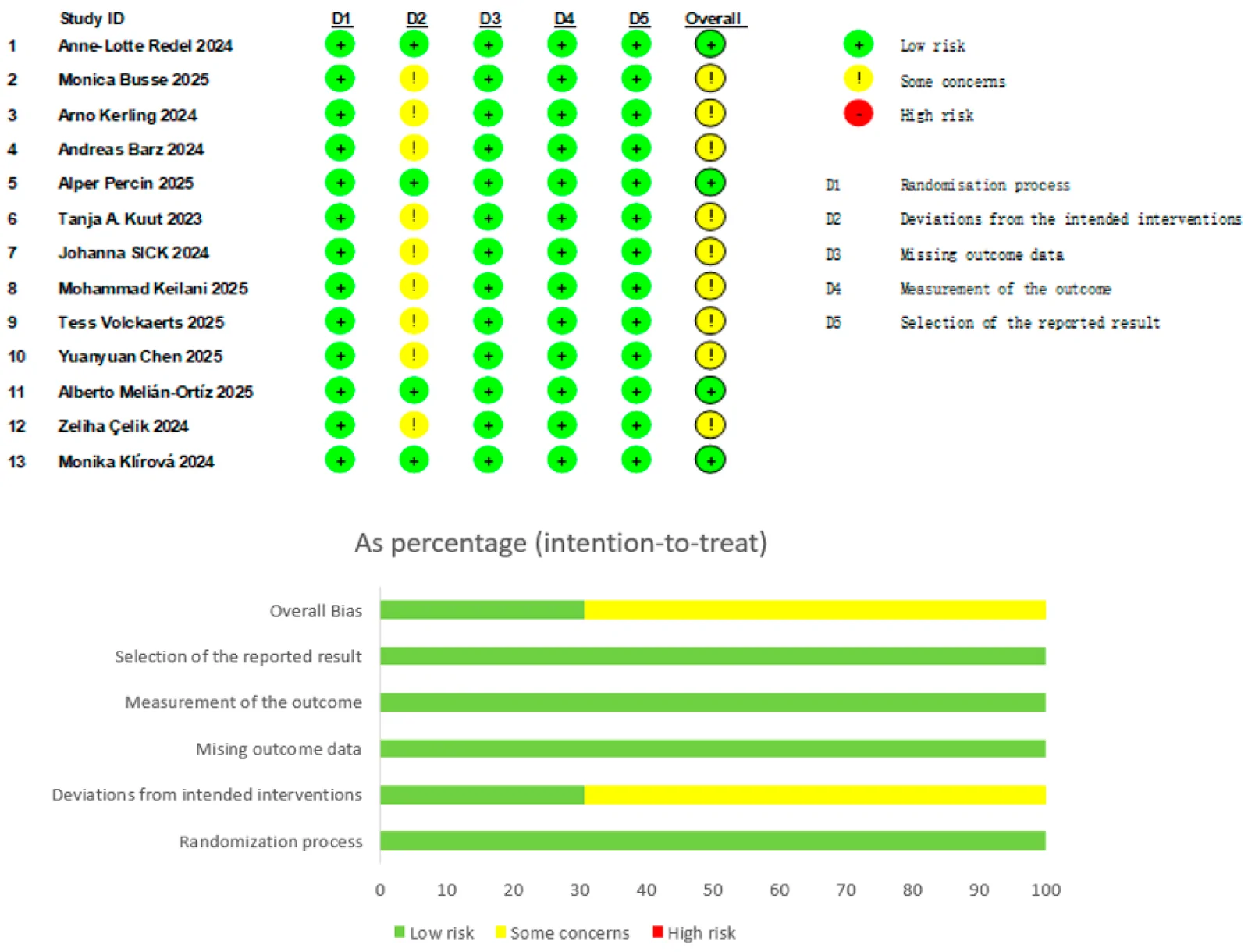
Risk of bias assessment. Refs. [32,33,34,35,36,37,38,39,40,41,42,43,44] review authors’ judgments about each risk of bias item presented as percentages across all included studies.

Risk of bias was evaluated using the RoB 2 tool, and the findings are presented in Figure 2 (File S3). Inter-rater agreement between the two reviewers was high, with a Cohen’s kappa coefficient of 0.83, indicating almost agreement. All 13 studies were judged to have a low risk of bias for the randomisation process. They employed adequate randomisation techniques (e.g., computer-generated sequences, block randomisation, and minimisation) and appropriate allocation concealment measures (e.g., sealed opaque envelopes and centralised web-based randomisation).

Regarding deviations from intended interventions, four studies that employed a rigorous double-blind design and conducted analyses following the intention-to-treat (ITT) principle were rated as low risk, with no evidence of bias introduced by deviations from the intended interventions. The remaining nine were judged as having some concerns, primarily due to incomplete blinding, meaning that the possibility of unblinding during the trial could not be fully excluded.

All studies had a low risk of bias in the domain of missing outcome data. They clearly reported the number, reasons, and distribution of dropouts and losses to follow-up, with overall attrition below 15% in most trials, and applied appropriate statistical approaches (e.g., ITT analysis, multiple imputation) to handle missing data.

For the measurement of the outcome domain, all studies were judged to have a low risk of bias. Although fatigue was assessed using self-reported instruments and participants were not always blinded to intervention assignment, the outcome measurements were based on prespecified, validated fatigue instruments that were applied consistently across intervention and control groups at clearly specified assessment time points. The lack of participant blinding was considered in the risk-of-bias assessment but was not, in itself, considered sufficient to indicate a high risk of bias in outcome measurement. Finally, all studies were considered low risk for selective reporting, as they were prospectively registered on relevant clinical trial registries and fully reported all pre-specified outcomes from the study protocols.

### 3.4. Effects of Non-Pharmacological Interventions on Fatigue

**Figure 3:**
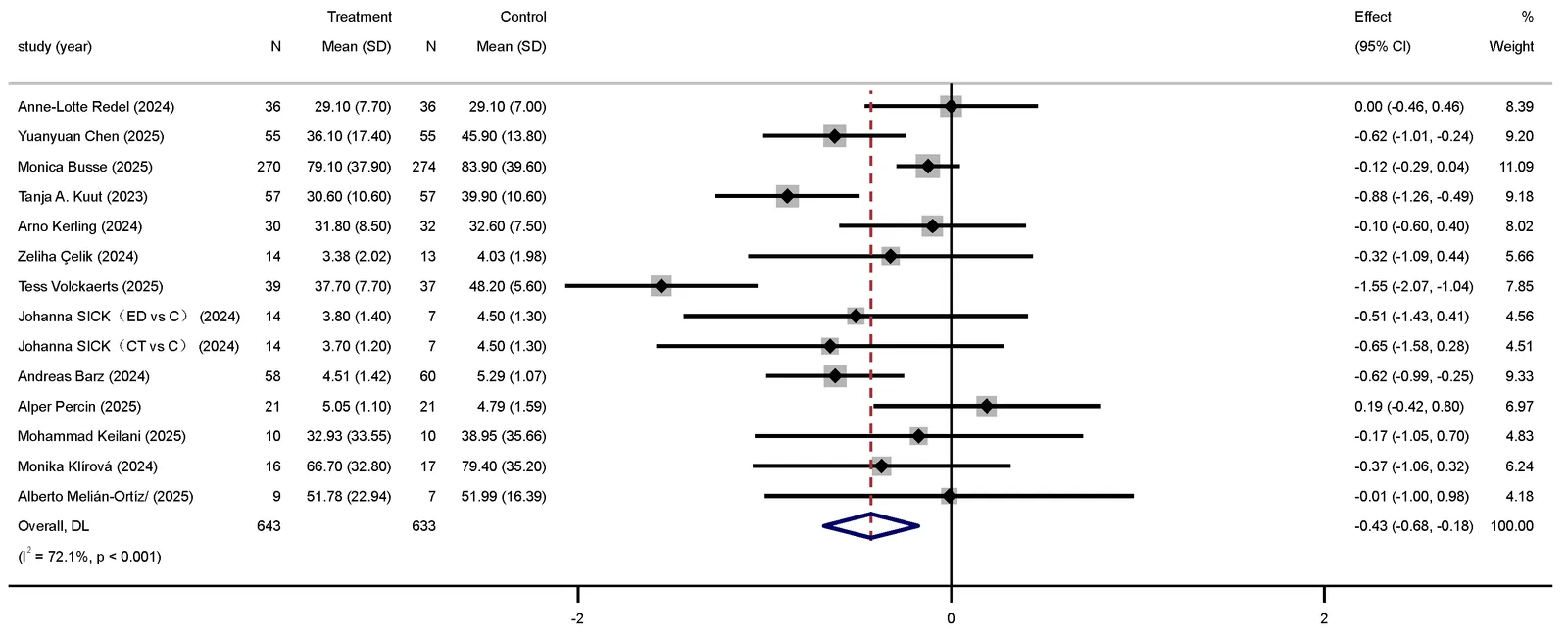
Forest plot of the effects of non-pharmacological interventions on fatigue. A random-effects model was used [32,33,34,35,36,37,38,39,40,41,42,43,44].

All 13 RCTs (contributing to 14 intervention–control comparisons) provided eligible data for evaluating the effect of non-pharmacological interventions on fatigue in patients with long COVID. As shown in Figure 3, considerable heterogeneity was detected across studies (*I*^2^ = 72.1%, *p* < 0.001 for Cochran’s Q test); accordingly, a random-effects model was adopted for pooled analysis. The meta-analysis showed that non-pharmacological interventions significantly reduced the fatigue scores compared with the controls [SMD = −0.43, 95% CI −0.68 to −0.18, *p* < 0.001]. The 95% prediction interval for the pooled effect ranged from −1.315 to 0.456.

Although the included interventions differed in their therapeutic mechanisms, they were combined in the primary meta-analysis because they shared the common clinical objective of improving fatigue in long COVID. The appropriateness of this combination was supported by the exploratory subgroup analyses, which did not detect a statistically significant difference between intervention types (between-subgroup *p* = 0.286), suggesting that the heterogeneity across modalities was not primarily driven by the type of intervention.

### 3.5. Subgroup Analyses

Because substantial heterogeneity was observed in the primary analysis, we conducted exploratory subgroup analyses to examine whether the pooled effect differed according to intervention type, intervention duration, and fatigue assessment instrument. These subgroup analyses were not pre-specified in the registered protocol and were therefore considered exploratory. As one included trial was a three-arm study, this methodological handling has been detailed in the Section 2.5. For clarity, the counts reported in subgroup analyses refer to the number of effect sizes (i.e., intervention-versus-control comparisons), not the number of studies; accordingly, the total across subgroups (14) may exceed the number of included studies (13).

#### 3.5.1. Subgroup Analysis by Intervention Type

**Figure 4:**
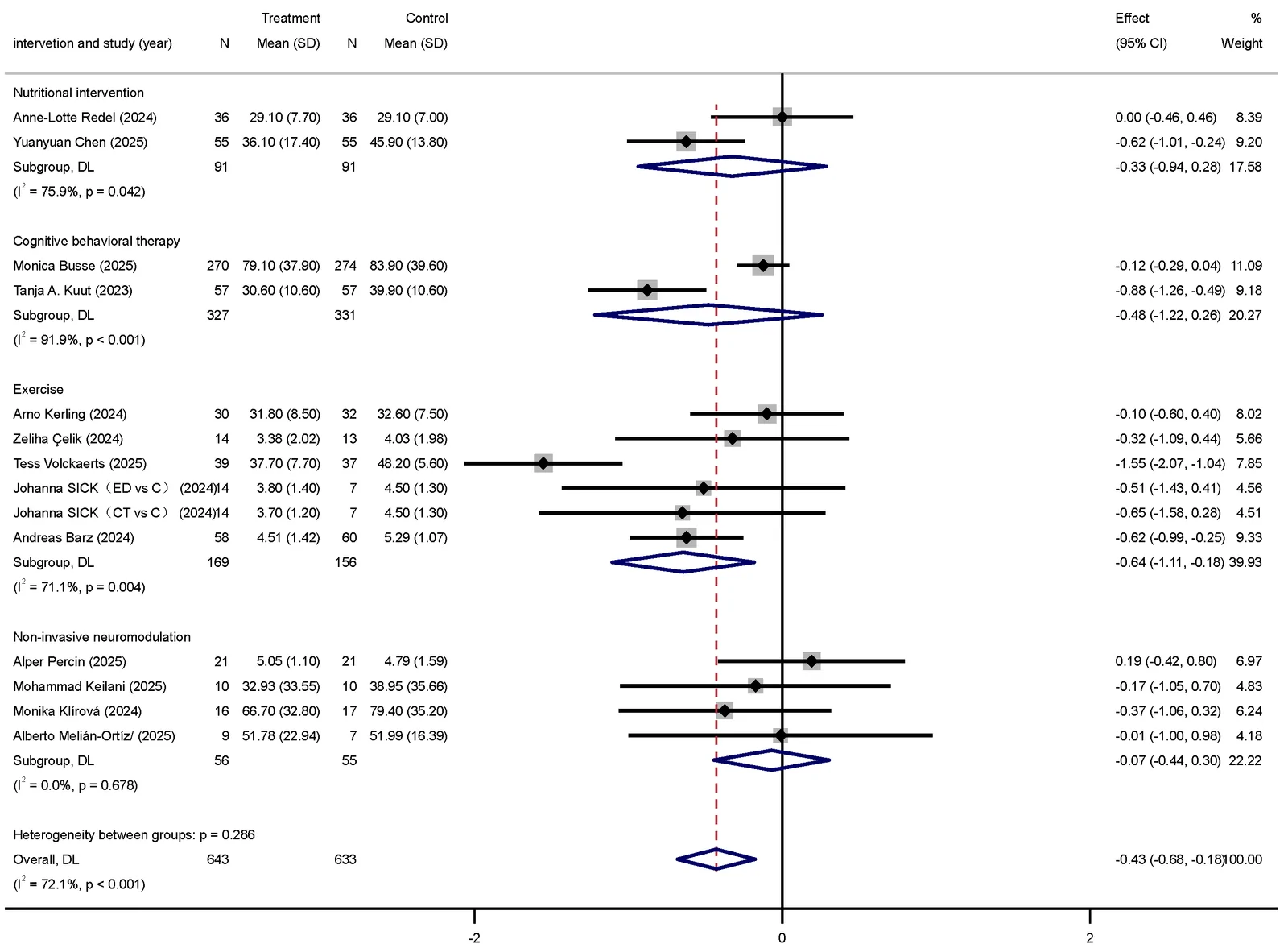
Subgroup analysis of effects by intervention modality on fatigue [32,33,34,35,36,37,38,39,40,41,42,43,44].

Subgroup analyses stratified by intervention type are presented in Figure 4. The nutritional intervention subgroup (*n* = 2) showed no significant effect [SMD = −0.33, 95% confidence interval (CI): −0.94 to 0.28, *p* > 0.05], with considerable heterogeneity (*I*^2^ = 75.9%, *p* = 0.042). The cognitive behavioural therapy subgroup (*n* = 2) yielded no statistically significant pooled effect [SMD = −0.48, 95% CI: −1.22 to 0.26, *p* > 0.05], with high heterogeneity (*I*^2^ = 91.9%, *p* < 0.001). The exercise intervention subgroup (*n* = 6) demonstrated a significant reduction in fatigue scores [SMD = −0.64, 95% CI: −1.11 to −0.18, *p* = 0.006], with moderate heterogeneity (*I*^2^ = 71.1%, *p* = 0.004). The non-invasive neuromodulation subgroup (*n* = 4) showed no significant effect [SMD = −0.07, 95% CI: −0.44 to 0.30, *p* > 0.05], with no heterogeneity detected (*I*^2^ = 0.0%, *p* = 0.678).

The between subgroup difference was not statistically significant (*p* = 0.286). However, given the small number of studies in the cognitive behavioural therapy (*n* = 2) and nutritional supplementation (*n* = 2) subgroups, these subgroup results should be interpreted as exploratory and hypothesis-generating. The absence of a significant subgroup difference does not exclude the possibility that intervention type may influence treatment effects; larger head-to-head trials are needed to evaluate comparative efficacy.

#### 3.5.2. Subgroup Analysis by Intervention Duration

**Figure 5:**
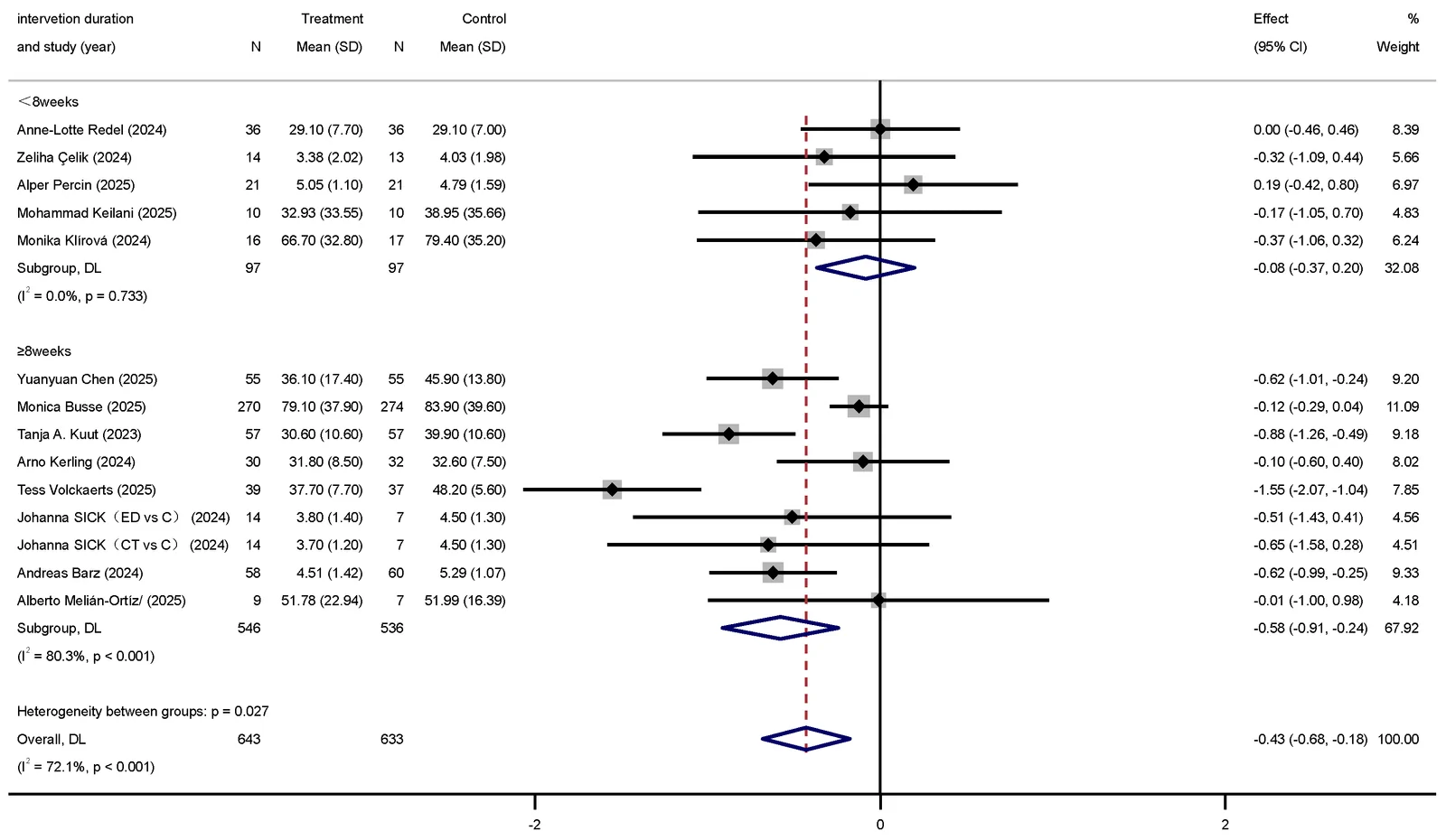
Subgroup analysis by intervention duration for fatigue outcomes [32,33,34,35,36,37,38,39,40,41,42,43,44].

Subgroup analyses stratified by intervention duration are presented in Figure 5. The subgroup with intervention duration < 8 weeks (*n* = 5) had no statistically significant heterogeneity (*I*^2^ = 0%, *p* = 0.733), with no significant between-group difference [SMD = −0.08 (95% CI: −0.37 to 0.20), *p* > 0.05]. The subgroup with intervention duration ≥ 8 weeks (*n* = 9) demonstrated considerable heterogeneity (*I*^2^ = 80.3%, *p* < 0.001), with a significant reduction in fatigue scores in the intervention group [SMD = −0.58 (95% CI: −0.91 to −0.24), *p* < 0.05].

The between-subgroup difference was statistically significant (*p* = 0.027), suggesting that intervention duration may be a source of heterogeneity. However, because longer-duration interventions were disproportionately represented by exercise trials in this meta-analysis, this finding should be interpreted with caution; the observed duration-related benefit cannot be definitively disentangled from the effect of intervention modality.

#### 3.5.3. Subgroup Analysis by Fatigue Assessment Instrument

**Figure 6:**
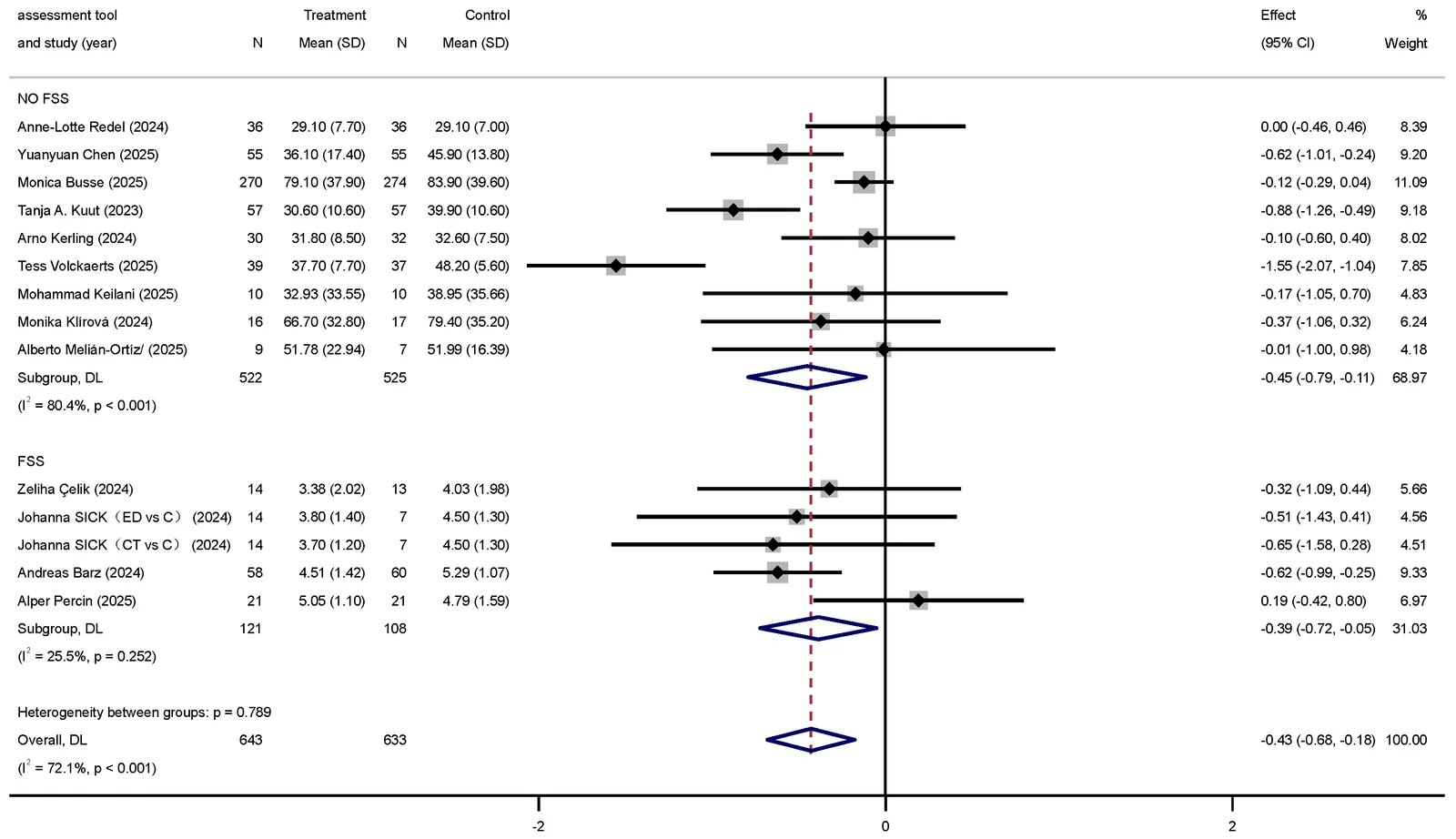
Subgroup analysis of effects on fatigue stratified by fatigue assessment instrument [32,33,34,35,36,37,38,39,40,41,42,43,44].

Subgroup analyses stratified by fatigue assessment instrument are presented in Figure 6. In the FSS subgroup (*n* = 5), heterogeneity was low (*I*^2^ = 25.5%, *p* = 0.252), and the pooled results showed that fatigue scores in the intervention group were significantly lower than those in the control group [SMD = −0.39 (95% CI: −0.72 to −0.05), *p* < 0.05]. In the non-FSS subgroup (*n* = 9), considerable heterogeneity was observed (*I*^2^ = 80.4%, *p* < 0.001), with the intervention group still showing significantly lower fatigue scores [SMD = −0.45 (95% CI: −0.79 to −0.11), *p* < 0.05]. However, given the considerable heterogeneity and the diversity of assessment instruments included in this subgroup, this pooled estimate should be interpreted with caution.

The between-subgroup difference was not statistically significant (*p* = 0.789), indicating that the choice of fatigue assessment instrument did not substantially alter the pooled effect estimates. Although heterogeneity was lower in the FSS subgroup (*I*^2^ = 25.5%) than in the non-FSS subgroup (*I*^2^ = 80.4%), this difference in heterogeneity should not be interpreted as evidence that the FSS is a more sensitive or reliable instrument. The non-FSS subgroup comprised diverse scales measuring related but distinct constructs (e.g., FIS, MFI, BFI), which may have contributed to the higher heterogeneity. No firm conclusions about the comparative performance of different fatigue scales can be drawn from these explorations.

### 3.6. Assessment of Publication Bias and Sensitivity Analysis

**Figure 7:**
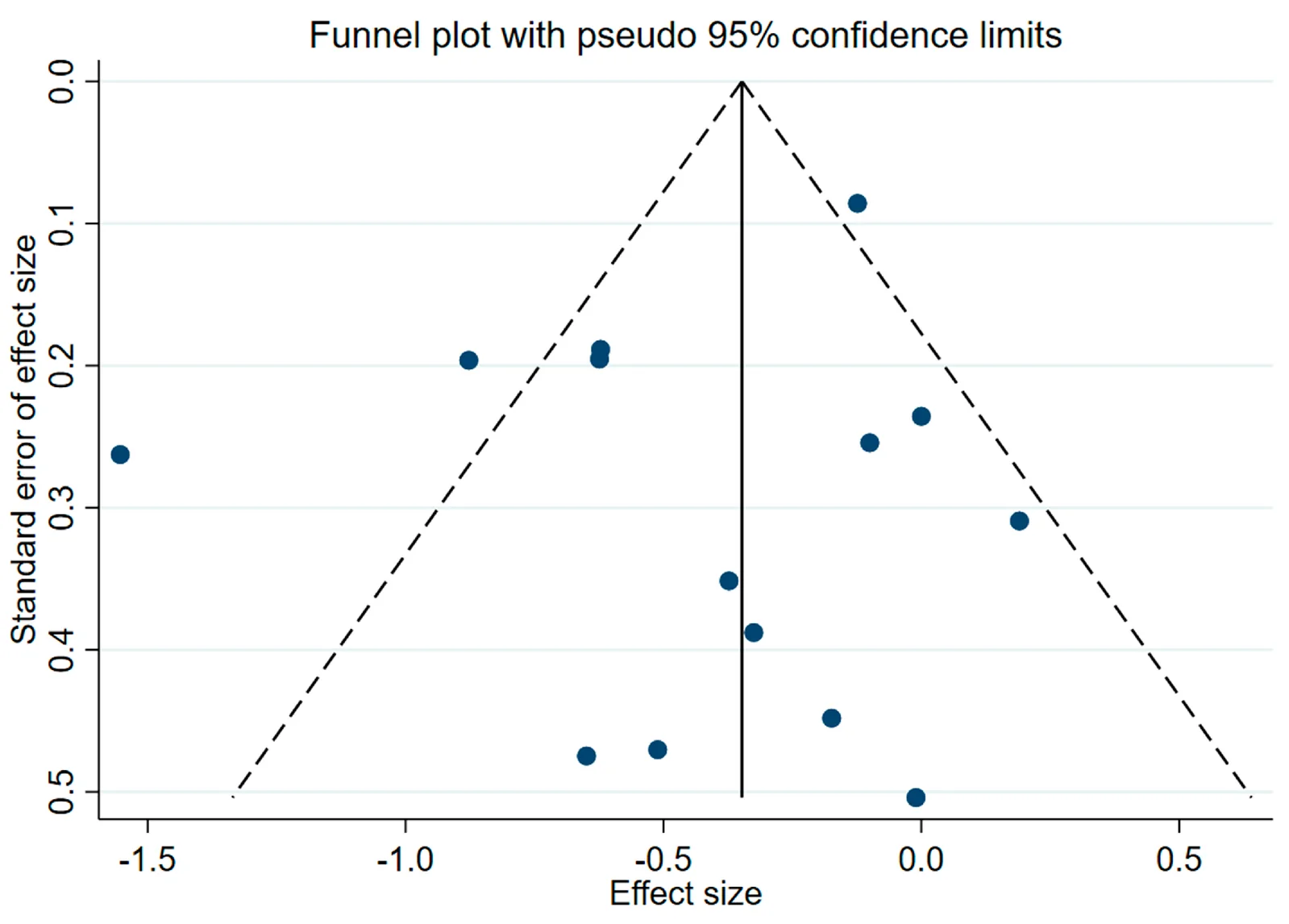
Funnel plot for the assessment of publication bias.

Publication bias was evaluated using a funnel plot (Figure 7), which was based on all 14 intervention–control comparisons derived from the 13 included studies, and appeared roughly symmetrical. Egger’s test was not statistically significant (*p* = 0.747), suggesting that small-study effects and publication bias were unlikely to be present. The trim-and-fill method identified no missing studies (imputed = 0), and the adjusted pooled effect size remained unchanged, further supporting this conclusion.

**Figure 8:**
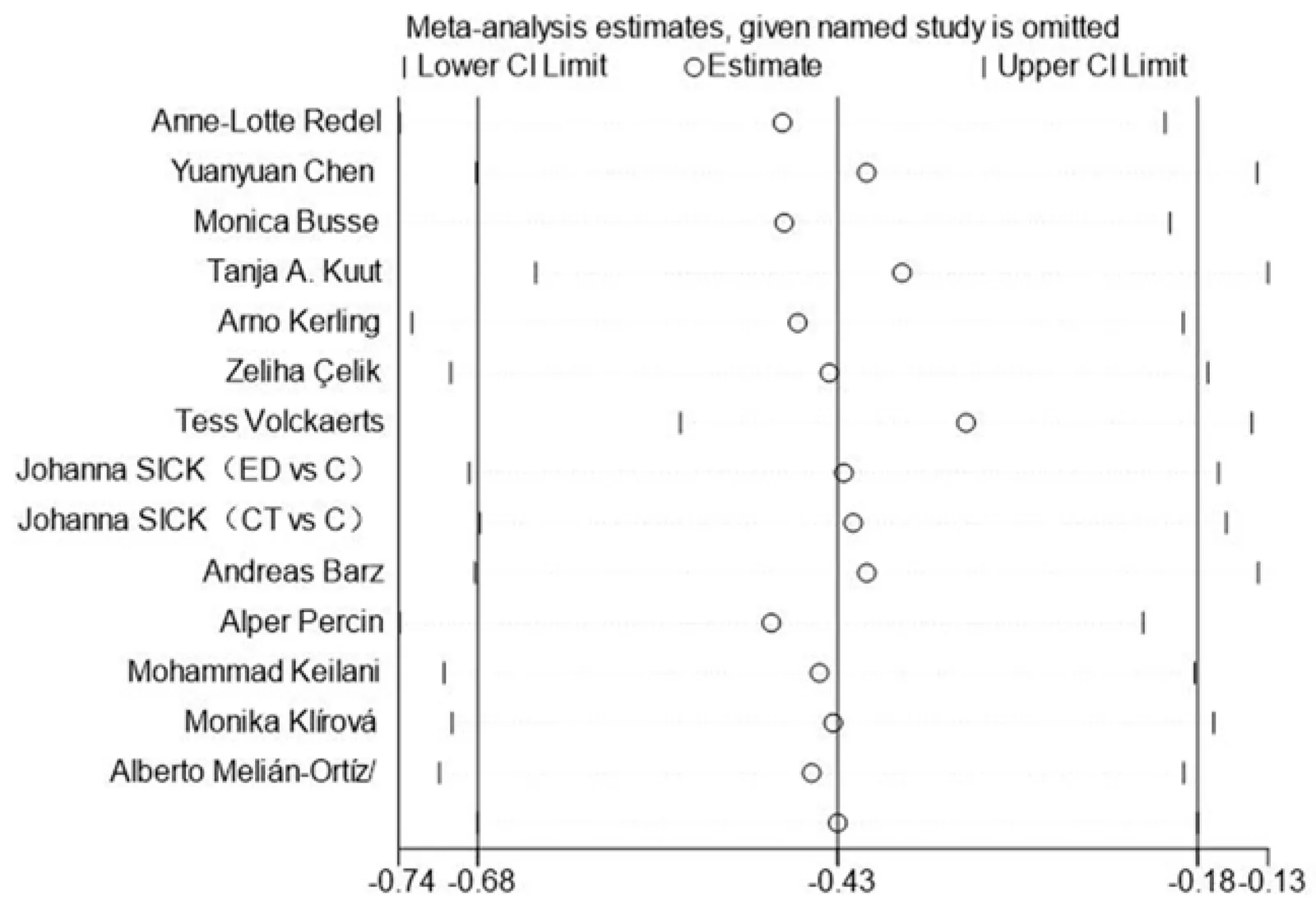
Sensitivity analysis plot [32,33,34,35,36,37,38,39,40,41,42,43,44].

To test the robustness of the pooled estimates, we performed a leave-one-out sensitivity analysis to assess the impact of each intervention–control comparison on the overall result (Figure 8). Sequential omission of each intervention–control comparison did not materially alter the magnitude or direction of the pooled SMD, indicating that the overall findings were reasonably robust.

## 4. Discussion

We synthesised evidence from 13 randomized controlled trials in this systematic review and meta-analysis, evaluating the effects of multiple non-pharmacological interventions on fatigue in patients with long COVID. The pooled analysis demonstrated that non-pharmacological interventions significantly reduced fatigue severity. Subgroup analyses further revealed that exercise training was the only modality associated with a statistically significant reduction in fatigue, and interventions lasting ≥8 weeks were associated with greater improvements; however, other intervention modalities did not show statistically significant pooled effects. Although the magnitude and consistency of effects varied by intervention type and duration, these findings suggest that non-pharmacological interventions may offer a feasible approach to alleviating fatigue in patients with long COVID. Nevertheless, cautious interpretation is warranted, as the 95% prediction interval was −1.315 to 0.456, which crossed the null and reflects the considerable between-study heterogeneity (*I*^2^ = 72.1%). This underscores that the pooled average effect may not be generalizable to all individual patients or future trial settings.

This study further enriches the evidence base for non-pharmacological interventions targeting fatigue symptoms in patients with long COVID. Existing meta-analyses have largely focused on single intervention modalities such as exercise training and pulmonary rehabilitation, with most evidence supporting their benefits for fatigue reduction [45,46,47]; however, these reviews are generally restricted to a single type of intervention and lack a systematic [48], comprehensive assessment of diverse non-pharmacological approaches. By incorporating the most recently published randomized controlled trials [33,36,38,39,40,41,42], the present study systematically evaluated the efficacy of four categories of non-pharmacological interventions: exercise training, cognitive behavioural therapy, non-invasive neuromodulation, and nutritional supplements. We conducted exploratory subgroup analyses to explore potential sources of heterogeneity, thereby offering a more comprehensive evidence base for the integrated management of long-COVID fatigue. Notably, statistically significant benefits were observed only in the exercise intervention subgroup. However, the between-subgroup difference was not statistically significant (*p* = 0.286), meaning that we cannot conclude that exercise is superior to other non-pharmacological interventions. The non-significant findings for cognitive behavioural therapy, non-invasive neuromodulation, and nutritional supplementation should be interpreted with caution; they are more likely attributable to the limited number of studies and small sample sizes in these subgroups than to a true absence of efficacy. Thus, the current evidence supports exercise training as the modality with the most data, but not necessarily as the most effective intervention. Given the multidimensional nature of long-COVID fatigue, future rehabilitation strategies may need to integrate motor, respiratory, cognitive, and behavioural components rather than relying on isolated interventions. Emerging evidence from dual-task and augmented reality-based rehabilitation provides preliminary support for this multimodal approach [12,49]; however, its additional benefits over single-modality interventions require confirmation through future randomized controlled trials.

Subgroup analyses suggested a trend toward differential effects, with only the exercise training subgroup demonstrating a statistically significant improvement. Several interconnected mechanisms have been proposed to explain the benefit of exercise, including improved aerobic fitness, enhanced mitochondrial function, rebalancing of autonomic tone, and reduced systemic inflammation—all of which are plausibly linked to the pathophysiology of long-COVID fatigue [11,50,51]. Psychosocial pathways, such as improved sleep quality, reduced anxiety, and enhanced self-efficacy, may also contribute [49,52]. However, none of these mechanisms were directly evaluated in this meta-analysis, and the relative contribution of each remains speculative. The observed effect should therefore be interpreted as an association, not as proof of causal mechanism. Considerable variability existed within the exercise subgroup in terms of intervention modality (e.g., aerobic training alone, resistance training, and combined programmes), exercise intensity (low-intensity vs. moderate-to-high-intensity), and supervision format (in-person supervision, remote monitoring, and home-based unsupervised training). These factors may have collectively contributed to the substantial heterogeneity observed in this subgroup. However, owing to the limited number of included studies, further stratified analyses by these dimensions were not feasible. Therefore, the optimal exercise prescription for long-COVID fatigue—including the most appropriate combination of modality, intensity, frequency, and supervision mode—remains to be established through well-designed head-to-head randomized controlled trials.

Although exercise training showed the largest pooled effect among the included modalities, several factors should be considered when translating this finding into clinical practice. First, safety requires cautious interpretation. While most trials did not report serious adverse events directly attributed to the intervention, symptom worsening or post-exertional malaise (PEM) was documented in studies. These observations indicate that exercise is not uniformly tolerated and that symptom-titrated, individualized approaches are essential, particularly for patients with prominent PEM or post-exertional symptom exacerbation (PESE). Second, adherence and acceptability varied across studies. Adherence rates, where reported, ranged from approximately 78.5% to 95%, suggesting that supervised or remotely guided programmes may be acceptable to many patients. However, adherence was not consistently reported, and reasons for non-adherence were not systematically documented. Acceptability was rarely formally assessed using validated instruments, limiting our understanding of patient preferences and barriers to participation. Future implementation should incorporate the routine monitoring of adherence and patient-reported acceptability to inform service design. Third, we did not formally assess certainty using the GRADE framework, but the substantial between-study heterogeneity (*I*^2^ = 72.1%), the exploratory nature of the subgroup analyses, the variability in intervention protocols, and the limited number of RCTs for non-exercise modalities all reduce confidence in the pooled estimates. Therefore, while exercise training represents the most extensively studied modality, the available evidence does not support a strong, definitive recommendation for its superiority or safety over other non-pharmacological approaches in all patients with long COVID.

For cognitive behavioural therapy, non-invasive neuromodulation, and nutritional supplementation, this study did not observe a statistically significant pooled effect. This negative result can be interpreted from three levels. First, methodological inadequacy. The number of studies and sample sizes included in each subgroup were limited, resulting in low statistical power, and most studies had wide 95% confidence intervals for effect sizes, indicating substantial random error. If these interventions only confer a modest clinical benefit, the current sample sizes may be insufficient to detect the true effect, posing a risk of false-negative findings. Second, high heterogeneity of the intervention protocols. Within the same category, different studies varied considerably in intervention parameters, delivery modes, and treatment durations, lacking standardized protocols. Such clinical heterogeneity may dilute any potential real efficacy and is also an important reason for the non-significant pooled result. Third, limited true efficacy at the biological level. Although these interventions have been shown to be effective in other chronic fatigue populations, such as cancer-related fatigue and multiple-sclerosis-related fatigue [53,54], the pathophysiological mechanisms of long-COVID fatigue are specific, involving multiple pathways including viral persistence, chronic inflammation, autonomic dysfunction, and mitochondrial abnormalities [55]. Interventions targeting a single pathway may not be able to cover the complex pathophysiology of long-COVID fatigue; therefore, the possibility cannot be excluded that these interventions have only weak efficacy or even lack clinically meaningful benefit for the long-COVID population.

A key finding of this study is that intervention duration was the only identified effect modifier that accounted for part of the between-study heterogeneity. Interventions lasting ≥8 weeks yielded significant improvements in fatigue, whereas those with a duration of <8 weeks did not reach statistically significant benefits. This finding aligns with core principles of rehabilitation medicine: sustained physiological adaptations—whether in cardiorespiratory fitness, mitochondrial function, or skeletal muscle remodelling—require sufficient time to develop [19,56]; likewise, improvements in patients’ behavioural patterns, self-management capacity, and psychological status typically take time to emerge [57]. Accordingly, longer intervention courses are more likely to produce cumulative therapeutic effects. However, because long-duration interventions were disproportionately represented by exercise trials in this meta-analysis, we cannot definitively disentangle the independent effect of treatment duration from that of exercise modality. Thus, the observed duration-related benefit should be interpreted with caution, and future factorial-design randomized controlled trials are required to isolate the specific contribution of each component.

The test for between-subgroup differences by fatigue assessment instrument was not statistically significant (*p* = 0.789), suggesting that the choice of fatigue scale did not substantially influence the pooled effect estimates. This finding indicates that despite differences in measurement dimensions and scoring systems across scales, the estimated treatment effects remained consistent across different measurement instruments, thereby enhancing the robustness and generalisability of our conclusions across assessment settings [58]. However, the discrepancy in within-subgroup heterogeneity warrants attention: the FSS subgroup exhibited substantially lower heterogeneity (*I*^2^ = 25.5%) than the non-FSS subgroup (*I*^2^ = 80.4%), suggesting that the FSS may be a more consistent and reliable measure for detecting treatment-related changes in long-COVID fatigue. The FSS specifically focuses on the extent to which fatigue limits daily functional activities—a domain that lies at the core of patients’ clinical concerns and represents the defining feature of long-COVID fatigue [59,60]—which may explain its greater ability to capture clinically meaningful improvements and its lower between-study variability. Based on these findings, future trials of non-pharmacological interventions for long-COVID fatigue should consider prioritising the FSS as a core outcome measure. Adoption of a standardised instrument would improve cross-study comparability and facilitate more robust quantitative synthesis in future meta-analyses.

This study suggests that non-pharmacological interventions significantly alleviate fatigue in patients with long COVID (SMD = −0.43), with exercise training showing the largest point estimate among the subgroups. Given the current evidence, exercise-centred non-pharmacological interventions may be considered as part of long-COVID fatigue management, particularly for patients with persistent fatigue and functional limitations. However, for patients who experience post-exertional symptom exacerbation (PESE), exercise prescription must be approached with caution. Graded load progression, close symptom monitoring, and individualized pacing strategies are essential to avoid symptom deterioration. The optimal exercise type, intensity, frequency, and supervision mode remain unclear, and the recommendations provided here should be adapted to each patient’s tolerance and baseline functional status. This cautious approach is consistent with current clinical guidance for managing PESE in long COVID. For patients with comorbid emotional distress and activity avoidance behaviours, cognitive-behavioural strategies may be added as an adjunct to exercise regimens to produce synergistic improvements in fatigue symptoms and functional status. Patients should be encouraged to maintain regular long-term intervention and to select appropriate modalities based on individual tolerance.

This study has several limitations that should be considered. First, the number of included randomized controlled trials was limited (*n* = 13), and several subgroups contained only a small number of studies—specifically, only two studies each for cognitive behavioural therapy and nutritional supplementation—resulting in limited pooled sample sizes and reduced statistical power, which may have compromised the robustness and stability of subgroup effect estimates. The subgroup analyses involving these modalities were underpowered, and the null findings should not be interpreted as definitive evidence of lack of efficacy. We did not perform meta-regression to further quantify the contribution of potential effect modifiers. This decision was based on two considerations: (1) the limited number of included studies (*n* = 13), which renders meta-regression prone to overfitting and inflated Type I error rates; (2) several key covariates of interest, including mean age and disease duration, were not consistently reported across the included studies, precluding a complete-case meta-regression analysis without substantial loss of statistical power. Consequently, we adopted exploratory subgroup analyses as the primary approach to exploring heterogeneity, as this method requires only categorical moderators and is less sensitive to missing covariate data. We acknowledge that subgroup analyses may not fully capture all underlying drivers of between-study variation, and the small number of studies within certain subgroups (e.g., CBT, nutritional supplementation) limits the robustness of these exploratory findings. Second, considerable clinical heterogeneity existed among the included long COVID populations. Variations in symptom profiles, disease duration, and functional impairment may have influenced treatment responses and contributed to between-study heterogeneity. Therefore, the findings should be interpreted with caution when applied to specific long COVID phenotypes. Third, the diversity of fatigue assessment instruments posed challenges for result interpretation. To enable quantitative synthesis, we included studies using different fatigue scales and adopted the standardized mean difference (SMD) as the pooled effect measure. Although the FSS and non-FSS subgroups showed consistent directions of effect, the non-FSS subgroup comprised multiple scales (e.g., FIS, MFI, BFI) with distinct measurement dimensions and response sensitivity, contributing to substantial heterogeneity (*I*^2^ = 80.4%) and limiting the clinical interpretability of the pooled estimate for this subgroup. This variability in fatigue assessment instruments may have contributed to the observed statistical heterogeneity across studies. Fourth, substantial between-study heterogeneity was observed in the overall analysis (*I*^2^ = 72.1%). Despite exploratory subgroup analyses exploring heterogeneity by intervention type, duration, and assessment instrument, the limited number of included studies precluded further investigation into the effects of exercise parameters (type, intensity, frequency, supervision mode) and participant baseline characteristics (fatigue severity, disease duration, comorbidities) on treatment outcomes. As such, the sources of heterogeneity remain incompletely elucidated. Fifth, the 8-week threshold used in the duration subgroup analysis was a categorisation point chosen for the exploratory subgroup analysis rather than a data-driven, validated cut-off. Accordingly, the finding that interventions lasting ≥8 weeks yielded greater benefits should be considered exploratory rather than confirmatory, and should not be applied as an inflexible clinical benchmark. Sixth, in addition, the included studies varied substantially in how they monitored intervention delivery and adherence. Some trials used supervised sessions, while others relied on remote monitoring or self-report; adherence was not consistently reported across studies, limiting our ability to assess the relationship between intervention dose and outcomes. Acceptability was also rarely formally assessed. Future trials should systematically report adherence rates, monitoring procedures, and participant acceptability to inform real-world implementation. The 95% prediction interval was −1.315 to 0.456, which crossed the null and reflects the considerable between-study heterogeneity (*I*^2^ = 72.1%). This underscores that the pooled average effect may not be generalizable to all individual patients or future trial settings. Finally, reporting quality varied across the included studies, with some providing insufficient descriptions of randomisation methods, incomplete documentation of intervention protocols, and inadequate reporting of reasons for attrition. Overall risk of bias was judged as either “low risk” or “some concerns” across all studies, with no study rated at high risk; nevertheless, the potential for residual methodological bias cannot be entirely excluded. We did not formally assess the certainty of evidence using the GRADE framework. Given the substantial heterogeneity and the exploratory nature of the subgroup analyses, the overall certainty of the evidence for the pooled effect is low to moderate. Future high-quality randomized controlled trials are needed to increase confidence in these estimates.

Future studies should focus on large-scale, multicentre randomized controlled trials that adopt standardised diagnostic criteria for long COVID, uniform intervention protocols, and consistent core outcome measures to improve cross-study comparability. Future trials should also investigate integrated rehabilitation programmes combining exercise training, cognitive rehabilitation, dual-task interventions, virtual or augmented reality technologies, and respiratory rehabilitation, while determining whether multimodal approaches provide superior outcomes compared with single-modality interventions. Furthermore, treatment strategies should be tailored according to specific clinical phenotypes of long-COVID fatigue, and precision rehabilitation approaches targeting distinct pathophysiological subtypes should be further explored to advance personalised care. Head-to-head comparisons between different non-pharmacological interventions are needed to clarify their comparative efficacy and inform clinical prioritisation. Finally, long-term follow-up studies are essential to evaluate the sustained effects of interventions on functional recovery, quality of life, symptom relapse, and cost-effectiveness.

## 5. Conclusions

This synthesis of RCT evidence suggests that non-pharmacological interventions, particularly exercise-based programmes, may be beneficial for reducing fatigue severity in individuals with long COVID. Interventions lasting ≥8 weeks were associated with greater average improvements in exploratory subgroup analyses; however, this finding should not be interpreted as a minimum clinical threshold or a definitive recommendation, as the analysis was exploratory and the 8-week cut-off was not validated. Importantly, the observed average benefit does not guarantee improvement for every individual, and exercise prescription must be individualized. For patients with post-exertional symptom exacerbation, graded progression and close symptom monitoring are essential to avoid worsening of symptoms. The overall certainty of the current evidence is low to moderate due to substantial clinical and methodological heterogeneity, variability in intervention protocols, and the limited number of RCTs for non-exercise interventions. No definitive conclusions can be drawn regarding the comparative efficacy of different non-pharmacological interventions based on the available data. Future large-scale, high-quality RCTs with standardised protocols, consistent outcome measures, and long-term follow-up are needed to establish the efficacy, safety, and sustained benefits of specific interventions, and to inform individualised rehabilitation strategies for long-COVID fatigue.

## Data Availability

No new data were created or analyzed in this study. Data sharing is not applicable to this article.

## Supplementary Materials

The following supporting information can be downloaded at: https://www.mdpi.com/article/10.3390/healthcare14203483/s1, File S1: PRISMA 2020 checklist; File S2: Definitions of intervention modalities [61,62,63,64]; File S3: Excel results from the ROB2 literature quality assessment tool; File S4: Search queries used in different databases.
