# Supplementary material for: Effectiveness of Non-Pharmacological Interventions for Fatigue in Long COVID: A Systematic Review and Meta-Analysis of Randomized Controlled Trials
Source: Healthcare (Basel). 2026 Oct 16;14(20):3483. doi: 10.3390/healthcare14203483 (PMC13704095; doi:10.3390/healthcare14203483)
Supplement: Supplementary file 1 [file healthcare-14-03483-s001.zip › File S2 Definitions of intervention modalities.pdf]

Type of therapy	Definition
Exercise therapy	We operationally defined exercise as planned, structured, and repetitive bodily movement performed to improve or maintain one or more components of physical fitness[1]. The exercise therapies included in this study covered multiple exercise modalities, such as aerobic exercise, resistance exercise, and combined exercise, among others.
Cognitive behavior therapy (CBT)	CBT is an evidence-based psychological interventionaimed at evaluating, challenging, and modifying apatient's dysfunctional beliefs[2]. therapistmostly emphasizes homework assignments and outside-of-session activities with topics to cope with stressfulsituations.
Nutritional intervention	Nutritional interventions include micronutrients, amino acids, multinutrient formulations, microbiota-targeted therapies, and whole-diet patterns that may alleviate long COVID fatigue by targeting inflammation and gut dysbiosis[3].
Non-invasive neuromodulation	Non-invasive neuromodulation encompasses techniques that modulate neural activity through either central or peripheral pathways, including non-invasive brain stimulation (e.g., transcranial magnetic stimulation [TMS] and transcranial electrical stimulation [tES]) and peripheral nerve stimulation modalities (e.g., non-invasive vagus nerve stimulation [nVNS], supraorbital transcutaneous stimulation [STS], remote electrical neuromodulation [REN], and external trigeminal nerve stimulation [e-TNS])[4].
Control group	The control group received placebo, usual care, waitlist, or no intervention

References

[1] CASPERSEN C J, POWELL K E, CHRISTENSON G M. Physical activity, exercise, and physical fitness: definitions and distinctions for health-related research [J]. Public health reports (Washington, DC : 1974), 1985, 100(2): 126-131.

[2] THOMA N, PILECKI B, MCKAY D. Contemporary Cognitive Behavior Therapy: A Review of Theory, History, and Evidence [J]. Psychodynamic psychiatry, 2015, 43(3): 423-461.

[3] BIGMAN G, RUSU M E, SHELAWALA N, et al. A Comprehensive Scoping Review on Diet and Nutrition in Relation to Long COVID-19 Symptoms and Recovery [J]. Nutrients, 2025, 17(11).

[4] KE D, CHEN M, DAN X, et al. Effectiveness of neuromodulation techniques for migraine prophylaxis: a systematic review and network meta-analysis of randomized controlled trials [J]. BMC Complementary Medicine and Therapies, 2026, 26(1): 74.
