# Supplementary material for: Effectiveness of Non-Pharmacological Interventions for Fatigue in Long COVID: A Systematic Review and Meta-Analysis of Randomized Controlled Trials
Source: Healthcare (Basel). 2026 Oct 16;14(20):3483. doi: 10.3390/healthcare14203483 (PMC13704095; doi:10.3390/healthcare14203483)
Supplement: Supplementary file 1 [file healthcare-14-03483-s001.zip › File S4 Search queries used in different databases..pdf]

**Pubmed:**

| Number | Search terms | Results |
|---|---|---|
| **#1** | ((((((((((((Post-Acute COVID-19 Syndrome) OR (Long COVID)) OR (Long Haul COVID-19)) OR (Long-Haul COVID)) OR (PASC Post Acute Sequelae of COVID-19)) OR (Post Acute COVID-19 Syndrome)) OR (Post COVID-19 Syndrome)) OR (Post-Acute COVID-19 Sequelae)) OR (Post-Acute Sequelae of COVID-19)) OR (Post-Acute Sequelae of SARS-CoV-2 Infection)) OR (Post-COVID Conditions)) OR (Post-COVID-19 Syndrome)) | 46,180 |
| **#2** | ((Fatigue) OR (Lassitude))) | 169,659 |
| **#3** | #1 AND #2 | 4,003 |
| **#4** | "Randomized Controlled Trial" [Publication Type] | 654,802 |
| **#5** | #3 AND #4 | 92 |

**Web of Science:**

| Number | Search terms | Results |
|---|---|---|
| **#1** | **Post-Acute COVID-19 Syndrome** (Topic) or **Long COVID** (All Fields) or **Long Haul COVID-19** (All Fields) or **Long-Haul COVID** (All Fields) or **PASC Post Acute Sequelae of COVID-19** (All Fields) or **Post Acute COVID-19 Syndrome** (All Fields) or **Post COVID-19 Syndrome** (All Fields) or **Post-Acute COVID-19 Sequelae** (All Fields) or **Post-Acute Sequelae of COVID-19** (All Fields) or **Post-Acute Sequelae of SARS-CoV-2 Infection** (All Fields) or **Post-COVID Conditions** (All Fields) or **Post-COVID-19 Syndrome** (All Fields) | 58，371 |
| **#2** | **Fatigue** (Topic) or **Lassitude** (All Fields) | 352,375 |
| **#3** | (#1) AND #2 | 3731 |
| **#4** | **Randomized Controlled Trial** (Topic) | 587,488 |
| **#5** | **(#3) AND #4** | 241 |

**Embase:**

| Number | Search terms | Results |
|---|---|---|
| **#1** | 'long covid'/exp | 14,059 |
| **#2** | 'chronic covid syndrome':ab,ti OR 'chronic covid-19':ab,ti OR 'covid long-hauler':ab,ti OR 'covid-19 long-hauler':ab,ti OR 'long haul covid':ab,ti OR 'long haul covid-19':ab,ti OR 'long hauler covid':ab,ti OR 'post covid 19 fatigue':ab,ti OR 'post covid 19 neurological syndrome':ab,ti OR 'post covid 19 syndrome':ab,ti OR 'post covid fatigue':ab,ti OR 'post covid impairment':ab,ti OR 'post covid syndrome':ab,ti OR 'post-acute covid syndrome':ab,ti OR 'post-acute covid-19':ab,ti OR 'post-acute covid-19 fatigue':ab,ti OR 'post-acute covid-19 neurological syndrome':ab,ti OR 'post-acute covid-19 syndrome':ab,ti OR 'post-acute sequelae of sars-cov-2 infection':ab,ti OR 'post-covid condition':ab,ti OR 'post-covid-19 condition':ab,ti OR 'long covid':ab,ti | 14,611 |
| **#3** | #2 OR #1 | 18,151 |
| **#4** | 'fatigue'/exp | 424,079 |

| | | |
|---|---|---|
| **#5** | tiredness:ab,ti OR fatigue:ab,ti | 276,677 |
| **#6** | #4 OR #5 | 480,973 |
| **#7** | #3 AND #6 | 15,285 |
| **#8** | 'randomized controlled trial'/exp | 1,220,343 |
| **#9** | 'controlled trial, randomized':ab,ti OR 'randomised controlled study':ab,ti OR 'randomised controlled trial':ab,ti OR 'randomized controlled study':ab,ti OR 'trial, randomized controlled':ab,ti OR 'randomized controlled trial':ab,ti | 313,954 |
| **#10** | #6 OR #7 | 1,268,639 |
| **#11** | #8 AND #10 | 918 |

**Cochrane:**

| Number | Search terms | Results |
|---|---|---|
| **#1** | MeSH descriptor: [Post-Acute COVID-19 Syndrome] explode all trees | 438 |
| **#2** | (Long COVID):ti,ab,kw OR (Post-Acute COVID-19 Sequelae):ti,ab,kw OR (Long Haul COVID 19):ti,ab,kw OR (Long Haul COVID-19s):ti,ab,kw OR (COVID, Long-Haul):ti,ab,kw (Word variations have been searched) | 3，067 |
| **#3** | (Long-Haul COVID):ti,ab,kw OR (Long Haul COVID):ti,ab,kw OR (Post Acute COVID-19 Syndrome):ti,ab,kw OR (Post-COVID Condition):ti,ab,kw OR (COVID-19 Syndrome, Post-Acute):ti,ab,kw (Word variations have been searched) | 1，594 |
| **#4** | (Post Acute Sequelae of SARS CoV2 Infection):ti,ab,kw OR (Post Acute COVID 19 Syndrome):ti,ab,kw OR (PASC Post Acute Sequelae of COVID 19):ti,ab,kw OR (Post-COVID-19 Syndrome):ti,ab,kw OR (PASC Post Acute Sequelae of COVID-19):ti,ab,kw (Word variations have been searched) | 1，309 |
| **#5** | (Post-Acute Sequelae of SARS-CoV-2 Infection):ti,ab,kw OR (Post-Acute COVID-19 Syndromes):ti,ab,kw OR (Post-COVID Conditions):ti,ab,kw OR (COVID-19, Long Haul):ti,ab,kw OR (Post Acute Sequelae of COVID 19):ti,ab,kw (Word variations have been searched) | 1，105 |
| **#6** | (Long Haul COVID-19):ti,ab,kw AND (Long-Haul COVIDs):ti,ab,kw AND (COVID-19 Post-Acute Sequelae):ti,ab,kw AND (Post-Acute Sequelae of COVID-19):ti,ab,kw AND (Post COVID Conditions):ti,ab,kw (Word variations have been searched) | 0 |
| **#7** | (Post COVID-19 Syndrome):ti,ab,kw (Word variations have been searched) | 1，507 |
| **#8** | #1 OR #2 OR #3 OR #4 OR #5 OR #6 OR #7 | 4，183 |
| **#9** | MeSH descriptor: [Fatigue] explode all trees | 6，693 |
| **#10** | (Lassitude):ti,ab,kw (Word variations have been searched) | 2，07 |
| **#11** | #9 OR #10 | 6，894 |
| **#12** | (Randomized Controlled Trial):ti,ab,kw (Word variations have been searched) | 977，609 |
| **#13** | #8 AND #11 AND #12 | 67 |
